# British Sign Language Recognition via Late Fusion of Computer Vision and Leap Motion with Transfer Learning to American Sign Language

**DOI:** 10.3390/s20185151

**Published:** 2020-09-09

**Authors:** Jordan J. Bird, Anikó Ekárt, Diego R. Faria

**Affiliations:** 1 ARVIS Lab—Aston Robotics Vision and Intelligent Systems, Aston University, Birmingham B4 7ET, UK; d.faria@aston.ac.uk; 2 School of Engineering and Applied Science, Aston University, Birmingham B4 7ET, UK; a.ekart@aston.ac.uk

**Keywords:** sign language recognition, multimodality, late fusion

## Abstract

In this work, we show that a late fusion approach to multimodality in sign language recognition improves the overall ability of the model in comparison to the singular approaches of image classification (88.14%) and Leap Motion data classification (72.73%). With a large synchronous dataset of 18 BSL gestures collected from multiple subjects, two deep neural networks are benchmarked and compared to derive a best topology for each. The Vision model is implemented by a Convolutional Neural Network and optimised Artificial Neural Network, and the Leap Motion model is implemented by an evolutionary search of Artificial Neural Network topology. Next, the two best networks are fused for synchronised processing, which results in a better overall result (94.44%) as complementary features are learnt in addition to the original task. The hypothesis is further supported by application of the three models to a set of completely unseen data where a multimodality approach achieves the best results relative to the single sensor method. When transfer learning with the weights trained via British Sign Language, all three models outperform standard random weight distribution when classifying American Sign Language (ASL), and the best model overall for ASL classification was the transfer learning multimodality approach, which scored 82.55% accuracy.

## 1. Introduction

Sign language is the ability to converse mainly by use of the hands, as well as in some cases the body, face and head. Recognition and understanding of Sign Language is thus an entirely visuo-temporal process performed by human beings. In the United Kingdom alone, there are 145,000 deaf adults and children who use British Sign Language (BSL) [1]. Of those people, 15,000 report BSL as their main language of communication [2], which implies a difficulty of communication with those who cannot interpret the language. Unfortunately, when another person cannot interpret sign language (of who are the vast majority), a serious language barrier is present due to disability.

In addition to the individuals who act as interpreters for those who can only converse in Sign Language, or who only feel comfortable doing so, this work aims to improve autonomous classification techniques towards dictation of Sign Language in real-time. The philosophy behind this work is based on a simple argument: If a building were to have a ramp in addition to stairs for easier access of the disabled, then why should a computer system not be present in order to aid with those hard of hearing or deaf? In this work, we initially benchmark two popular methods of sign language recognition with an RGB camera and a Leap Motion 3D hand tracking camera after gathering a large dataset of gestures. Following these initial experiments, we then present a multimodality approach which fuses the two forms of data in order to achieve better results for two main reasons: first, mistakes and anomalous data received by either sensor has the chance to be mitigated by the other, and second, a deep neural network can learn to extract useful complimentary data from each sensor as well as the standard approach of extracting information towards the class itself. The driving force behind improving the ability of these two sensors is mainly cost, in that the solution presented is of extremely minimal cost and, with further improvement beyond the 18 gestures explored in this study, could easily be implemented within public places such as restaurants, schools, libraries, etc. in order to improve the lives of disabled individuals and enable communication with those they otherwise could not communicate with.

In this work, the approaches of single modality learning and classification are compared to multimodality late fusion. The main scientific contributions presented by this work are as follows.

1. Collection of a large BSL dataset from five subjects and a medium-sized ASL dataset from two subjects (The dataset is publicly available at https://www.kaggle.com/birdy654/sign-language-recognition-leap-motion).
2. Tuning of classification models for the RGB camera (processing layer prior to output), Leap Motion Classification (evolutionary topology search) and multimodality late fusion of the two via concatenation to a neural layer. Findings show that multimodality is the strongest approach for BSL classification compared to the two single-modality inputs as well as state of the art statistical learning techniques.
3. Transfer learning from BSL to improve ASL classification. Findings show that weight transfer to the multimodality model is the strongest approach for ASL classification.

The remainder of this work is as follows. Section 2 explores the current state-of-the-art for Sign Language Classification. Section 3 details the method followed for these experiments, which includes data collection, data preprocessing and the machine learning pipeline followed. The results for all of the experiments are presented in Section 4, including indirect comparison to other state-of-the-art works in the field, before conclusions are drawn and future work is suggested in Section 5.

## 2. Related Work

Sign Language Recognition (SLR) is a collaboration of multiple fields of research which can involve pattern matching, computer vision, natural language processing and linguistics [3,4,5]. The core of SLR is often times focused around a feature engineering and learning model-based approach to recognising hand-shapes [6]. Classically, SLR was usually performed by temporal models trained on sequences of video. Many works from the late 1990s through to the mid-2000s found best results when applying varying forms of Hidden Markov Models (HMMs) to videos [7,8,9,10]; HMMs are predictive models of transition (prior distribution) and emission probabilities (conditional distribution) of hidden states. To give a specific example, researchers found in [7] that hand-tracking via a camera and classification of hand gestures while wearing solidly coloured gloves (similar to chroma key) was superior to hand-tracking without a glove. In this work, a vector of eight features was extracted from the hands including 2-dimensional X,Y positions, the angle of the axis of with the least inertia and the eccentricity of a bounding ellipse around the hand. That is, four features for each hand. These vectors then provided features as input to the HMM. More recently though, given affordable sensors that provide more useful information than a video clip, studies have focused upon introducing this information towards stronger and more robust real-time classification of non-verbal languages. Sign language recognition with depth-sensing cameras such as Kinect and Leap Motion is an exciting area within the field due to the possibility of accessing accurate 3D information from the hand through stereoscopy similar to human depth perception via images from two eyeballs. Kinect allows researchers to access RGBD channels via a single colour camera and a single infrared depth-sensing camera. A Microsoft Kinect camera was used to gather data in [11], and features were extracted using a Support Vector Machine from depth and motion profiles. Researchers in [12] found that generating synchronised colour-coded joint distance topographic descriptor and joint angle topographical descriptor and used as input to a two-steam CNN produced effective results; the CNNs in this study were concatenated by late fusion similar to the multimodality method in this study and results were ~92% for a 20-class dataset. In terms of RGB classification specifically, many state-of-the-art works have argued in favour of the VGG16 architecture [13] for hand gesture recognition towards sign language classification [14]. These works include British [15], American [16], Brazilian [17] and Bengali [18] Sign Languages, among others. Given the computational complexity of multimodality when visual methods are concerned in part, multimodality is a growing approach to hand gesture recognition. Researchers have shown that the approach of fusing the LMC and flexible sensors attached to the hand via Kalman filtering [19] is promising. Likewise in this regard, recent work has also shown that RGBD (Realsense) along with a physical sensor-endowed glove can also improve hand-tracking algorithms [20]. Given the nature of SLR, physically-worn devices are an unrealistic expectation for users to accept when real-world situations are considered, e.g., should someone wish to sign in a hotel lobby for staff who do not know sign language. For this reason, we follow the approach of two non-physical sensors that are placed in front of the subject as a “terminal”. That is, facing towards a camera and Leap Motion sensor are similar to natural social interaction and do not require the adoption of a physical device on the body.

Transfer Learning is a relatively new idea applied to the field of Sign Language recognition. In [21], researchers found it promising that knowledge could be transferred between a large text corpora and BSL via both LSTM and MLP methods, given that sign language data is often scarcely available. In this work, rather than transferring between syntax-annoted text corpora, we aim to follow the multisensor experiments with transfer learning between two different sign languages, i.e., transferring between the same task but in two entirely different languages (British Sign Language and American Sign Language).

**Figure 1:**
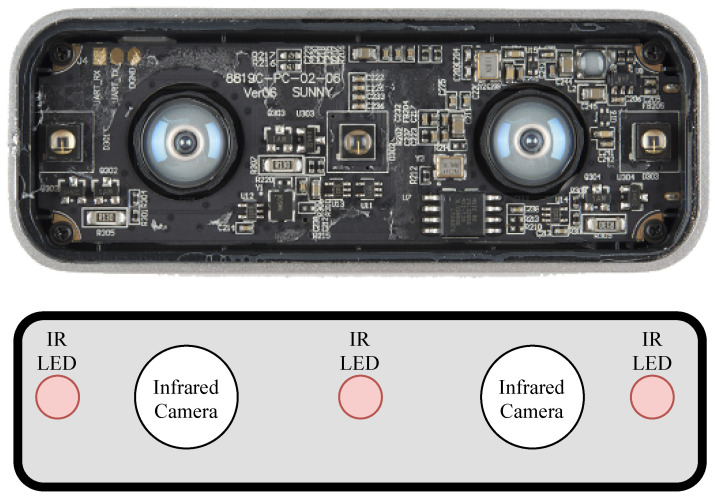
Photograph and labelled sketch of the stereoscopic infrared camera array within a Leap Motion Controller, illuminated by three infrared light-emitting diodes (IR LEDs).

**Figure 2:**
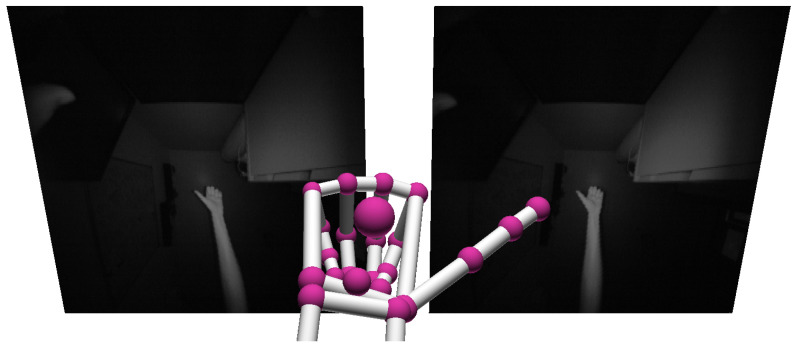
Screenshot of the view from Leap’s two infrared cameras and the detected hand reproduced in 3D. Note that this study uses a front-facing view rather than up-facing as shown in the screenshot.

The Leap Motion Controller, a sketch of which can be observed in Figure 1, is a device that combines stereoscopy and depth-sensing in order to accurately locate the individual bones and joints of the human hand. An example of the view of the two cameras translated to a 3D representation of the hand can be seen in Figure 2. The device measures 3.5 × 1.2 × 0.5 inches and is thus a more portable option compared to the Microsoft Kinect. Features recorded from the 26 letters of the alphabet in American Sign Language were observed to be classified at 79.83% accuracy by a Support Vector Machine algorithm [22]. Similarly to the aforementioned work, researchers found that a different dataset also consisting of 26 ASL letters were classifiable at 93.81% accuracy with a Deep Neural Network [23]. Another example achieved 96.15% with a deep learning approach on a limited set of 520 samples (20 per letter) [24]. Data fusion via Coupled Hidden Markov Models was performed in [25] between Leap Motion and Kinect, which achieved 90.8% accuracy on a set of 25 Indian Sign Language gestures.

Additionally, studies often fail to apply trained models to unseen data, and therefore towards real-time classification (the ultimate goal of SL recognition). With this in mind, Wang et al. proposed that sign language recognition systems are often affected by noise, which may negatively impact real-time recognition abilities [26]. In this work, we benchmark two single-modality approaches as well as a multimodality late fusion approach of the two both during training, and on unseen data towards benchmarking a more realistic real-time ability. Additionally, we also show that it is possible to perform transfer learning between two ethnologues with the proposed approaches for British and American Sign Languages.

In much of the state-of-the-art work in Sign Language recognition, a single modality approach is followed, with multimodality experiments being some of the latest studies in the field.

The inspiration for the network topology and method of fusion in this work comes from the work in [27] (albeit applied to scene recognition in this instance), similarly, this work fuses two differing synchronous data types via late-fusion by benchmarking network topologies at each step. In the aforementioned work however, weights of the networks were frozen for late fusion layer training (derived from benchmarking the two separate models). In this experiment, all weights are able to train from the start of the late fusion network from scratch, and thus the networks can extract complimentary features from each form of data for classification in addition to the usual method of extracting features for direct classification and prediction.

Table 1 shows a comparison of state-of-the-art approaches to Sign Language recognition. The training accuracy found in this work is given as comparison as other works report such metric, but it is worth noting that this work showed that classification of unseen data is often lower than the training process. For example, the multimodality approach score of 94.44% was reduced to 76.5% when being applied to completely unseen data.

## 3. Proposed Approach: Multimodality Late Fusion of Deep Networks

**Figure 3:**
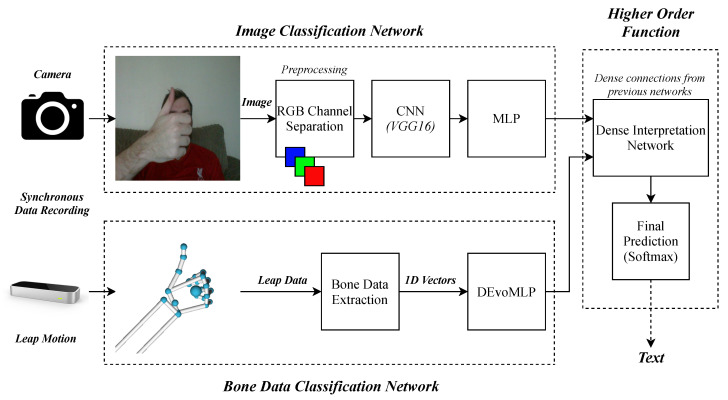
An overall diagram of the three benchmarking experiments. Above shows the process of image classification and below shows Leap Motion data classification for the same problem of sign language recognition. The higher order function network shows the late fusion of the two to form a multimodality solution.

Within this section, the proposed approach for the late fusion experiments is described. The experiments that this section mainly refers to can be observed in Figure 3, which outlines the image classification, Leap Motion classification and multimodality late fusion networks. The camera is used to record an image, and features are extracted via the VGG16 CNN and MLP. The Leap motion is used to record a numerical vector representing the 3D hand features previously described, which serves as input to an evolutionarily optimised deep MLP. Given that data is recorded synchronously, that is, the image from the camera and the numerical vector from the Leap Motion are captured at the same moment in time, the data objects are used as the two inputs to the multimodality network as they both describe the same frame captured.

### 3.1. Dataset Collection and Preprocessing

**Figure 4:**
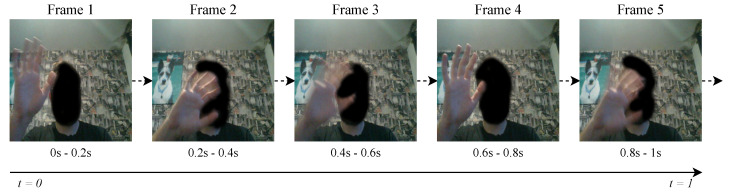
An example of one second of RGB image data collected at a frequency of 0.2 s per frame (5 Hz). Alongside each image that is taken is a numerical vector collected from the Leap Motion Controller.

Five subjects contributed to a dataset of British Sign Language, where each of the gestures was recorded for thirty seconds each, 15 s per dominant hand. Rather than specific execution times, subjects are requested to repeat the gesture at a comfortable speed for the duration of the recording; a recording of 15 s in length prevents fatigue from occurring and thus affecting the quality of the data. An example of recorded image data can be observed in Figure 4. Eighteen differing gestures were recorded at a frequency of 0.2 s each using a laptop, an image was captured using the laptop’s webcam and Leap Motion data are recorded from the device situated above the camera facing the subject. This allowed for “face-to-face” communication, as the subject was asked to communicate as if across from another human being. The “task-giver” was situated behind the laptop and stopped data recording if the subject made an error while performing the gesture. Each 0.2 s recording provides a data object that is inserted into the dataset as a numerical vector to be classified.

**Figure 5:**
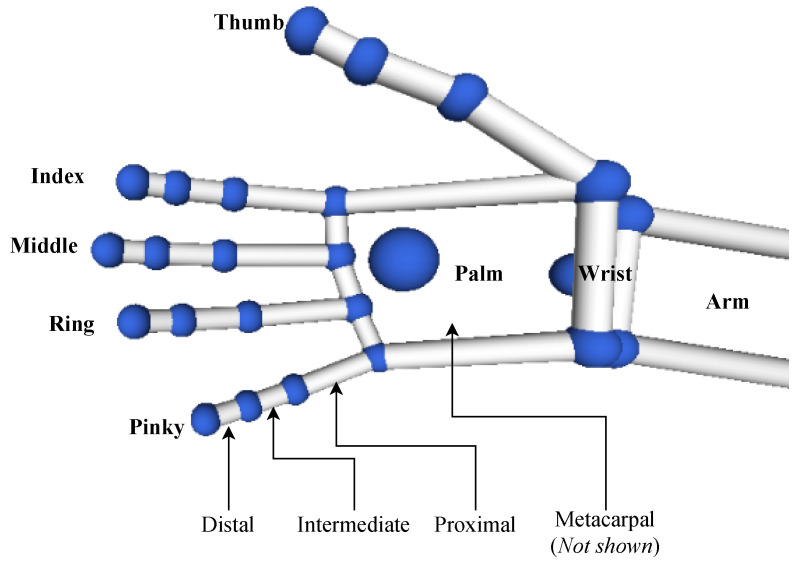
Labelled diagram of the bone data detected by the Leap Motion sensor. Metacarpal bones are not rendered by the LMC Visualiser.

From the Leap Motion sensor, data were recorded for each of the thumb, index, middle, ring and pinky fingers within the frame (labelled “left” or “right”). The names of the fingers and bones can be observed in the labelled diagram in Figure 5. For each hand, the start and end positions; 3D angles between start and end positions; and velocities of the arm, palm and finger bones (metacarpal, proximal, intermediate and distal bones) were recorded in order to numerically represent the gesture being performed. The pitch, yaw and roll of the hands were also recorded. If one of the two hands were not detected, then its values were recorded as “0” (e.g., a left handed action will also feature a vector of zeroes for the right hand). If the sensor did not detect either hand, data collection was automatically paused until the hands were detected in order to prevent empty frames. Thus, every 0.2 s, a numerical vector is output to describe the action of either one or two hands. The θ angle is computed using two 3D vectors by taking the inverse cosine of the dot product of the two vectors divided by the magnitudes of each vector, as shown below.

(1) $$\theta =arccos\left(\frac{ab}{|a||b|}\right),$$

where ∣a∣ and ∣b∣ are

(2) $$\begin{array}{c} |a|=\sqrt{{a_{x}}^{2}+{a_{y}}^{2}+{a_{z}}^{2}}\\ |b|=\sqrt{{b_{x}}^{2}+{b_{y}}^{2}+{b_{z}}^{2}}, \end{array}$$

with regards to the x,y and *z* co-ordinates of each point in space. The start and end points of each bone in the hand from the LMC are treated as the two points.

The following is a summary of each feature collected from the hierarchy of arm to finger joint.

- For each arm:
  - Start position of the arm (X, Y and Z)
  - End position of the arm (X, Y and Z)
  - 3D angle between start and end positions of the arm
  - Velocity of the arm (X, Y and Z)
- For each elbow:
  - Position of the elbow (X, Y and Z)
- For each wrist:
  - Position of the wrist (X, Y and Z)
- For each palm:
  - Pitch
  - Yaw
  - Roll
  - 3D angle of the palm
  - Position of the palm (X, Y and Z)
  Velocity of the palm (X, Y and Z)
  - Normal of the palm (X, Y and Z)
- For each finger:
  - Direction of the finger (X, Y and Z)
  - Position of the finger (X, Y and Z)
  - Velocity of the finger (X, Y and Z)
- For each finger joint:
  - Start position of the joint (X, Y and Z)
  - End position of the joint (X, Y and Z)
  - 3D angle of the joint
  - Direction of the finger (X, Y and Z)
  - Position of the joint (X, Y and Z)
  - Velocity of the joint (X, Y and Z)

Each feature was pre-processed via a minmax scaler between 0 (min) and 1 (max): $Feat=Feat_{std}\left(max-min\right)+min$, where $Feat_{std}=\left(\frac{Feat-Feat_{min}}{Feat_{max}-Feat_{min}}\right)$. Thus, each feature value is reduced to a value between 0 and 1. This was performed as it was observed that non-processed feature values caused issues for the model and often resulted in classification accuracy scores of only approximately 4%, showing a failure to generalise. The 18 British Sign Language (Visual examples of the BSL gestures can be viewed at https://www.british-sign.co.uk/british-sign-language/dictionary/) gestures recorded were selected due to them being common useful words or phrases in language. A mixture of one and two-handed gestures were chosen. Each gesture was recorded twice where subjects switched dominant hands.

The useful gestures for general conversation were

1. Hello/Goodbye
2. You/Yourself
3. Me/Myself
4. Name
5. Sorry
6. Good
7. Bad
8. Excuse Me
9. Thanks/Thank you
10. Time

The gestures for useful entities were

1. Airport
2. Bus
3. Car
4. Aeroplane
5. Taxi
6. Restaurant
7. Drink
8. Food

**Figure 6:**
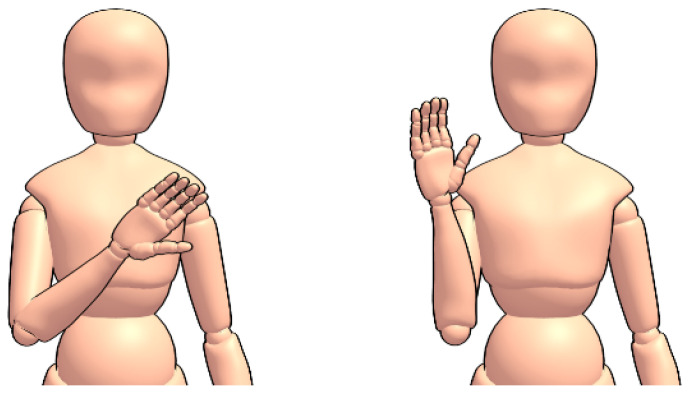
The sign for “Hello” in British Sign Language.

**Figure 7:**
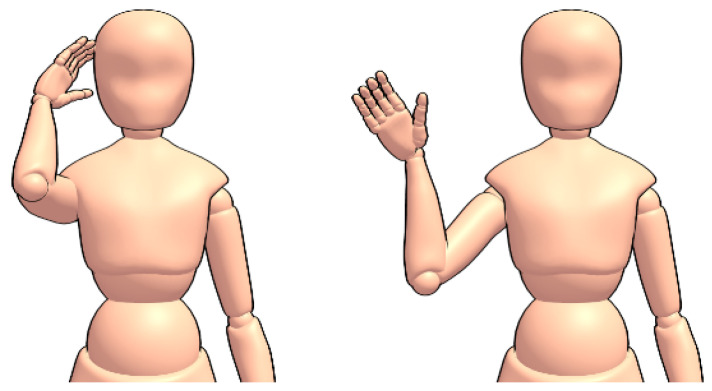
The sign for “Hello” in American Sign Language.

Following this, a smaller set of the same 18 gestures, but in American Sign Language (Visual examples of the ASL gestures can be viewed at https://www.handspeak.com/), are collected from two subjects for thirty seconds each (15 per hand) towards the transfer learning experiment. “Airport” and “Aeroplane/Airplane” in ASL are similar, and so “Airport” and “Jet Plane” are recorded instead. Figure 6 and Figure 7 show a comparison of how one signs “hello” in British and American sign languages; though the gestures differ, the hand is waved and as such it is likely that useful knowledge can be transferred between the two languages.

### 3.2. Deep Learning Approaches

**Figure 8:**
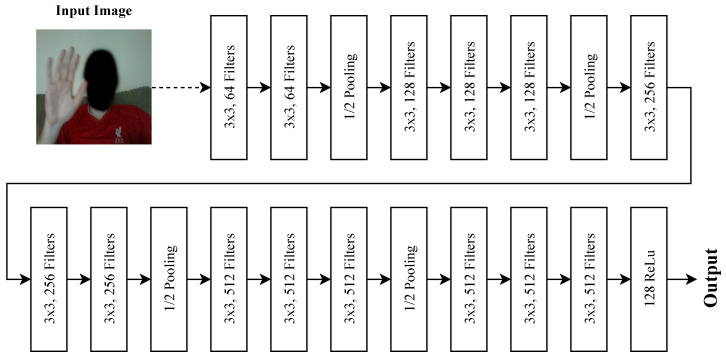
Feature extraction from the RGB branch of the network, the input image is passed through a fine-tuned VGG16 CNN [39] and then a layer of 128 ReLu neurons provide output. The network is trained via softmax output, but this softmax layer is later removed and the 128 outputs are used in late fusion with the Leap Motion network.

For the image classification network, VGG16 [39] convolutional layers are used as a starting point for feature extraction from image data, as can be seen in Figure 8, where the three 4096 neuron hidden layers are removed. The convolutional layers are followed by 2,4,8,…,4096 ReLu neuron layers in each of the ten benchmarking experiments to ascertain a best-performing interpretation layer. For the Leap Motion data classification problem, an evolutionary search is performed [40] to also ascertain a best-performing neural network topology; the search is set to a population of 20 for 15 generations, as during manual exploration, stabilisation of a final best result tends to occur at approximately generation 11. The evolutionary search is run three times in order to mitigate the risk of a local maxima being carried forward to the latter experiments.

With the best CNN and Leap Motion ANN networks derived, a third set of experiments is then run. The best topologies (with softmax layers removed) are fused into a single layer of ReLu neurons in the range 2,4,8,…,4096.

All experiments are benchmarked with randomised 10-fold cross-validation, and training time is uncapped to a number of epochs and rather executed until no improvement of accuracy occurs after 25 epochs. Thus, the results presented are the maximum results attainable by the network within this boundary of early stopping.

**Figure 9:**
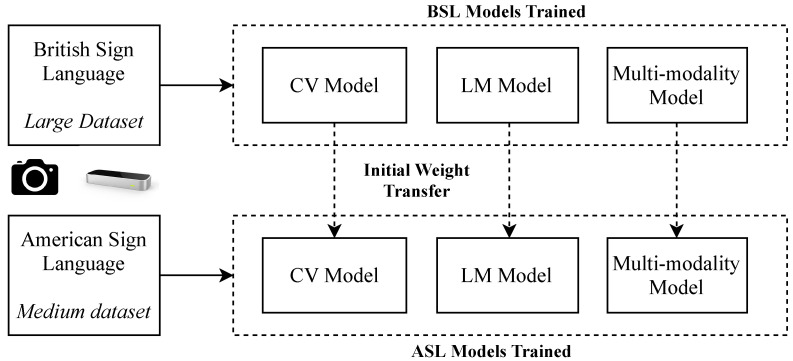
Transfer Learning Experiments which train on BSL and produce initial starting weight distributions for the ASL models.

Following the experiments on BSL, initial preliminary experiments for Transfer Learning between languages are performed. Figure 9 shows the outline for the transfer experiments, in which the learnt weights from the three BSL models are transferred to their ASL counterparts as initial starting weight distributions and ultimately compared to the usual method of beginning with a random distribution. This experiment is performed in order to benchmark whether there is useful knowledge to be transferred between each of the model pairs.

### 3.3. Experimental Software and Hardware

The deep learning experiments in this study were performed on an Nvidia GTX 980Ti which has 2816 CUDA cores (1190 MHz) and 6 GB of GDDR5 memory. Given the memory constraints, images are resized to 128 × 128 although they were initially captured in larger resolutions. All deep learning experiments were written in Python for the Keras [41] library and TensorFlow [42] backend.

The statistical models trained in this study were performed with a Coffee Lake Intel Core i7 at a clock speed of 3.7 GHz. All statistical learning experiments were written in Python for the SciKit-Learn library [43].

## 4. Experimental Results

### 4.1. Fine Tuning of VGG16 Weights and Interpretation Topology

**Figure 10:**
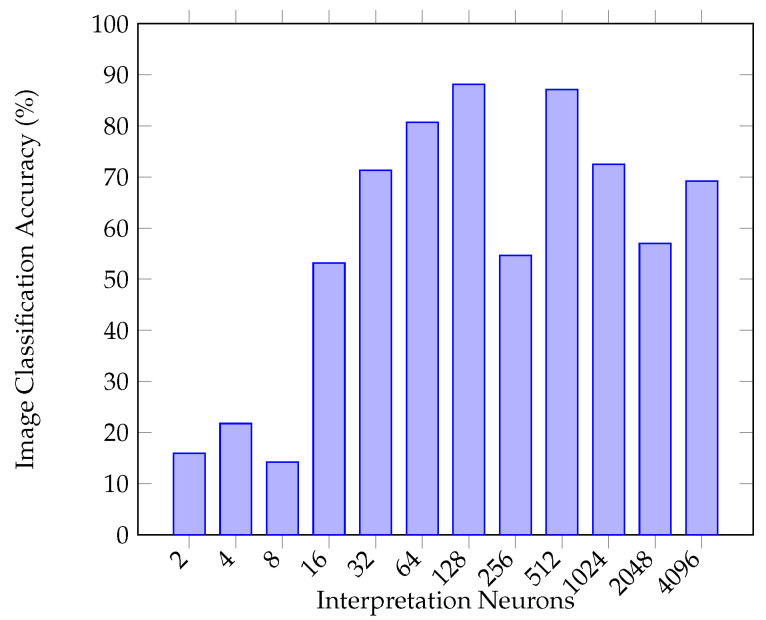
Mean image 10-fold classification accuracy corresponding to interpretation neuron numbers.

Figure 10 shows the results for tuning of the VGG network for image classification. Each result is given as the classification ability when a layer of neurons are introduced beyond the CNN operations and prior to output. The best result was a layer of 128 neurons prior to output, which resulted in a classification accuracy of 88.14%. Most of the results were relatively strong except for 2–8 neurons and, interestingly, layers of 256 and 2048 neurons. Thus, the CNN followed by 128 neurons forms the first branch of the multimodality system for image processing alongside the best Leap Motion network (in the next section). The SoftMax output layer is removed for purposes of concatenation, and the 128 neuron layer feeds into the interpretation layer prior to output.

### 4.2. Evolutionary Search of Leap Motion Dnn Topology

**Figure 11:**
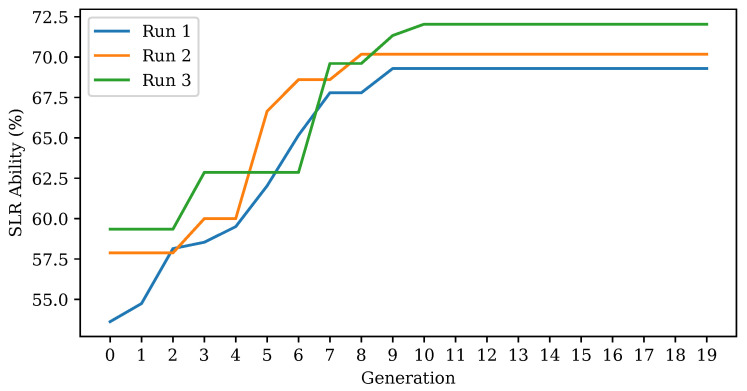
Three executions of optimisation of neural network topologies via an evolutionary algorithm.

The evolutionary search algorithm is applied three times for a population of 20 through 15 generations, which can be observed in Figure 11. The maximum number of neurons was 1024, and the maximum number of layers was 5. After an initial random initialisation of solutions, the algorithm performs roulette selection for each solution and generates an offspring (where number of layers and number of neurons per layer are bred). At the start of each new generation, the worst performing solutions outside of the population size 20 range are deleted and the process runs again. The final best result is reported at the end of the simulation. Table 2 shows the best results for three runs of the Leap Motion classification networks. Of the three, the best model was a deep neural network of 171,292,387 neurons which resulted in a classification accuracy of 72.73%. Interestingly, the most complex model found was actually the worst performing of the best three results selected. This forms the second branch of the multimodality network for Leap Motion classification in order to compliment the image processing network. Similarly to the image processing and network, the SoftMax output layer is removed and the final layer of 387 neurons for Leap Motion data classification is connected to the dense interpretation network layer along with the 128 hidden neurons of the image network. In terms of mean and standard deviations of the runs on a generational basis, Run 1 was 65.48% (5.37), Run 2 was 66.98% (4.87) and Run 3 was 68.02% (5.05). With regards to the mean and standard deviation of the three final results, they were 70.5% (1.14).

### 4.3. Fine-Tuning the Final Model

**Figure 12:**
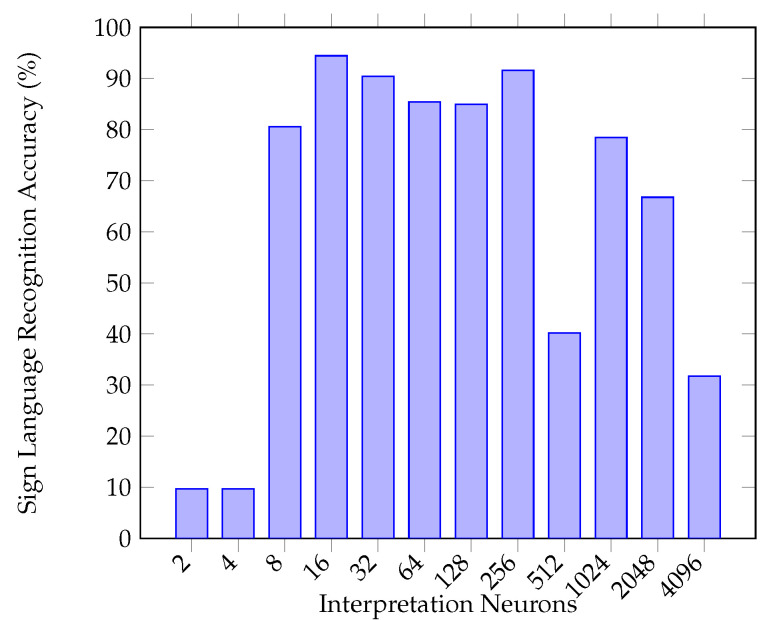
Multimodality 10-fold classification accuracy corresponding to interpretation neuron numbers towards benchmarking the late-fusion network.

Figure 12 shows the results of fine-tuning the best number of interpretation neurons within the late fusion layer; the best set of hyperparameters found to fuse the two prior networks was a layer of 16 neurons, which achieved an overall mean classification ability of 94.44%. This best-performing layer of 16 neurons receives input from the Image and Leap Motion classification networks and is connected to a final SoftMax output. Given the nature of backpropagation, the learning process enables the two input networks to perform as they were prior (that is, to extract features and classify data) but a new task is also then possible; to extract features and useful information from either data format which may compliment the other, for example, for correction of common errors, or for contributing to confidence behind a decision.

### 4.4. Comparison and Analysis of Models

Table 3 shows a comparison of the final three tuned model performances for recognition of British Sign Language through the classification of photographic images (RGB) and bone data (Leap Motion) compared to the multimodality approach that fuses the two networks together. The maximum classification accuracy of the CV model achieved 88.14%, the Leap Motion model achieved 72.73% but the fusion of the two allowed for a large increase towards 94.44% accuracy. A further comparison to other statistical approaches can be observed in Table 4, within which the different algorithms applied to the same dataset are shown and directly compared; although the DNN approach is relatively weak compared to all statistical models except for Gaussian Naive Bayes, it contributes to the multimodality approach by extracting features complimentary to the CNN prior to late fusion as well as the task of classification—this, in turn, leads to the multimodality approach attaining the best overall result. The best statistical model, the Random Forest, was outperformed by the CNN by 1.07% and the multimodality approach by 7.37%. Performance aside, it must be noted that the statistical approaches are far less computationally complex than deep learning approaches; should the host machine for the task not have access to a GPU with CUDA abilities, a single-modality statistical approach is likely the most realistic candidate. Should the host machine, on the other hand, have access to a physical or cloud-based GPU or TPU, then it would be possible to enable the most superior model, which was the deep learning multimodality approach.

Table 5 shows the ten highest scoring features gathered from the Leap Motion Controller by measure of their information gain or relative entropy. Right handed features are seemingly the most useful, which is possibly due to the most common dominant hand being the right. Though all features shown have relatively high values, it can be noted that the roll of the right hand is the most useful when it comes to classification of the dataset.

**Figure 13:**
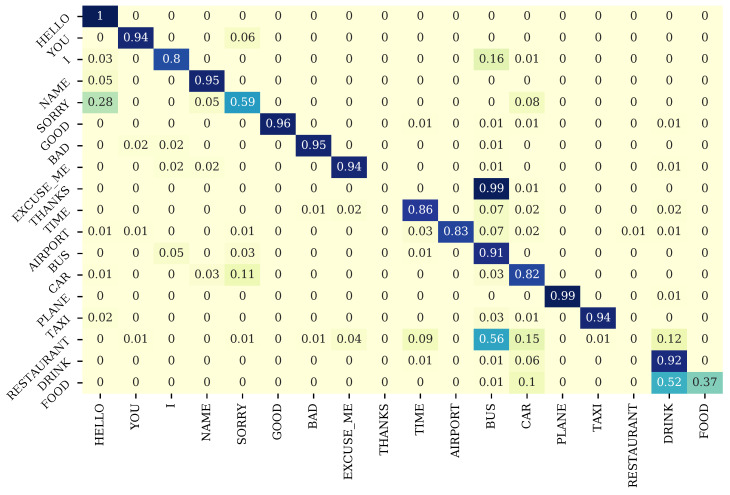
Confusion matrix for the best model (multimodality, 76.5%) on the set of unseen data (not present during training).

Table 6 shows the final comparison of all three models when tasked with predicting the class labels of unseen data objects (100 per class (18 classes)). The error matrix for the best model, which was the multimodality approach at 76.5% accuracy can be observed in Figure 13. Interestingly, most classes were classified with high confidence with the exception of three main outliers: “thanks” was misclassified as “bus” in almost all cases, “restaurant” was misclassified as a multitude of other classes and “food” was often mistaken for “drink”, although this did not occur vice versa. Outside of the anomalous classes which must be improved in the future with more training examples, the multimodality model was able to confidently classify the majority of all other phrases. Though it would require further experiments to pinpoint, it is likely that the poor performance of the leap motion suggests that such data is difficult to generalise outside of the learning process. Though, on the other hand, useful knowledge is still retained given the high accuracy of the multimodality model which considers it as input alongside a synchronised image.

### 4.5. Leave-One-Subject-Out Validation

Table 7 shows the training metrics for each model with a leave-one-subject-out approach. That is, training on all subjects but one, and then validation upon the left out subject. All models performed relatively well, with the interesting exception of the RGB camera when classifying subject 2, which scored only 68.24%. On average, the best approach remained the multimodality model, which scored 92.12% accuracy (+6.69% over RGB, +3.55% over Leap Motion). This finding is similar to the outcomes of the other experiments, where the multimodality model always outperformed the singular sensor approaches.

### 4.6. Transfer Learning from BSL to ASL

Table 8 shows the results for transfer learning from BSL to ASL. Interestingly, with the medium-sized ASL dataset and no transfer learning, the multimodality approach is worse than both the Computer Vision and Leap Motion models singularly. This, and considering that the best model overall for ASL classification was the multimodality model with weight transfer from the BSL model, suggests that data scarcity poses an issue for multimodality models for this problem.

The results show that transfer learning improves the abilities of the Leap Motion and multimodality classification approaches to sign language recognition. With this in mind, availability of trained weights may be useful to improve the classification of other datasets regardless of whether or not they are in the same sign language. Overall, the best model for ASL classification was the multimodality model when weights are transferred from the BSL model. This approach scored 82.55% classification ability on the ASL dataset. The results suggest that useful knowledge can be transferred between sign languages for image classification, Leap Motion classification and late-fusion of the two towards multimodality classification.

Though future work is needed for further explore the transfer learning hypotheses, the results in these initial experiments suggest the possibility of success when transferring knowledge between models and ultimately improving their recognition performances.

## 5. Conclusions and Future Work

This work has presented multiple experiments for the singular sensor and multimodality approaches to British and American Sign Language. The results from the experiments suggest that a multimodality approach outperforms the two singular sensors during both training and classification of unseen data. This work also presented a preliminary Transfer Learning experiment from the large BSL dataset to a medium-sized ASL dataset, in which the best model for classification of ASL was found to be the multimodality model when weights are transferred from the BSL model. All of the network topologies in this work that were trained, compared and ultimately fused together towards multimodality were benchmarked and studied for the first time. Accurate classification of Sign Language, especially unseen data, enables the ability to perform the task autonomously and thus provide a digital method to interpretation of non-spoken language within a situation where interpretation is required but unavailable. In order to realise this possibility, future work is needed. The hypotheses in these experiments were argued through a set of 18 common gestures in both British and American Sign Languages. In future, additional classes are required to allow for interpretation of conversations rather than the symbolic communication enabled by this study. In addition, as multimodality classification proved effective, further tuning of hyperparameters could enable better results, and other methods of data fusion could be explored in addition to the late fusion approach used in this work. Transfer learning could be explored with other forms of non-spoken language, for example, Indo-Pakistani SL, which has an ethnologue of 1.5 million people and Brazilian SL with an ethnologue of 200,000 people. The aim of this work was to explore the viability and ability of multimodality in Sign Language Recognition by comparing Leap Motion and RGB classification with their late-fusion counterpart. In addition, the 0.2s data collection frame poses a limitation to this study, and as such, further work could be performed to derive a best window length for data collection.

A cause for concern that was noted in this work was the reduction of ability when unseen data is considered, which is often the case in machine learning exercises. Such experiments and metrics (ability on unseen dataset and per-class abilities) are rarely performed and noted in the state-of-the-art works within sign language recognition. As the main goal of autonomous sign language recognition is to provide a users with a system which can aid those who otherwise may not have access to a method of translation and communication, it is important to consider how such a system would perform when using trained models to classify data that was not present in the training set. That is, real-time classification of data during usage of the system and subsequently the trained classification models. In this work, high training results were found for both modalities and multimodality, deriving abilities that are competitive when indirectly compared to the state of the art works in the field. When the best performing 94.44% classification ability model (multimodality) was applied to unseen data, it achieved 76.5% accuracy mainly due to confusion within the “thanks” and “restaurant” classes. Likewise, the RGB model reduced from 88.14% to 69.44% and the Leap Motion model reduced from 72.73% to 41.78% when comparing training accuracy and unseen data classification ability. Future work is needed to enable the models a better ability to generalise towards real-time classification abilities that closely resemble their abilities observed during training.

### Ethics

The requirements of the ethics procedure of Aston University are based on UK laws and were incorporated into the definition of the guidelines. All participants were informed in detail on the project characteristics and written informed consent was obtained. Special attention was given to the trial in order to ensure the compliance with ethical requirements, confidentiality and protection of personal data. No trials were performed without previous approval by the ethical committee and data protection based on the Ethics committee of the Aston University. All experiments were done in accordance with the highest ethical standards from the UK.

We ensured that the information given to the participants was easy to understand, and all written information was in accordance to the “Easy to Read” guidelines. All participants agreed with the “Informed Consent Form”.

## Figures and Tables

**Table 1:** Other state-of-the-art works in autonomous Sign Language Recognition, indirectly compared due to operation on different datasets and with different sensors. Note: It was observed in this study that classification of unseen data is often lower than results found during training, but many works do not benchmark this activity.

Study	Sensor	Input	Approach	Classes	Score (%)
Huang et al. [28]	Kinect	Skeleton	DNN	26	97.8
Filho et al. [29]	Kinect	Depth	KNN	200	96.31
Morales et al. [30]	Kinect	Depth	HMM	20	96.2
Hisham et al. [31]	LMC	Point Cloud	DTW	28	95
Kumar et al. [32]	LMC	Point Cloud	HMM, BLSTM	50	94.55
Quesada et al. [33]	RealSense	Skeleton	SVM	26	92.31
Kumar et al. [12]	MoCap	Skeleton	2-CNN	20	92.14
Yang [34]	Kinect	Depth	HCRF	24	90.4
Cao Dong et al. [35]	Kinect	Depth	RF	24	90
Elons et al. [36]	LMC	Point Cloud	MLP	50	88
Kumar et al. [37]	Kinect	Skeleton	HMM	30	83.77
Chansri et al. [38]	Kinect	RGB, Depth	HOG, ANN	42	80.05
Chuan et al. [22]	LMC	Point Cloud	SVM	26	79.83
Quesada et al. [33]	LMC	Skeleton	SVM	26	74.59
Chuan et al. [22]	LMC	Point Cloud	KNN	26	72.78
***This study***	LMC, RGB	Hand feats, RGB	CNN-MLP-LF	18	94.44

**Table 2:** Final results of the three evolutionary searches sorted by 10-fold validation accuracy along with the total number of connections within the network.

Hidden Neurons	Connections	Accuracy
171, 292, and 387	243,090	72.73%
57, 329, and 313	151,760	70.17%
309, 423, and 277	385,116	69.29%

**Table 3:** Sign Language Recognition scores of the three models trained on the dataset.

Model	Sign Language Recognition Ability
***RGB***	88.14%
***Leap Motion***	72.73%
***Multi-modality***	94.44%

**Table 4:** Comparison of other statistical models and the approaches presented in this work.; Deep Neural Network (DNN), Convolutional Neural Network (CNN), Random Forest (RF), Sequential Minimal Optimisation Support Vector Machine (SMO SVM), Quadratic Discriminant Analysis (QDA), Linear Discriminant Analysis (LDA), Logistic Regression (LR), and Naïve Bayes (NB).

Model	Input Sensor(s)	Sign Language Recognition Ability
MM(DNN, CNN)	LMC, Camera	94.44%
CNN	Camera	88.14%
RF	LMC	87.07%
SMO SVM	LMC	86.78%
QDA	LMC	85.46%
LDA	LMC	81.31%
LR	LMC	80.97%
Bayesian Net	LMC	73.48%
DNN	LMC	72.73%
Gaussian NB	LMC	34.91%

**Table 5:** The top ten features by relative entropy gathered from the Leap Motion Controller.

Leap Motion Feature	Information Gain (Relative Entropy)
***right_hand_roll***	0.8809
***right_index_metacarpal_end_x***	0.8034
***right_thumb_metacarpal_end_x***	0.8034
***right_pinky_metacarpal_end_x***	0.8034
***left_palm_position_x***	0.8033
***right_index_proximal_start_x***	0.8028
***left_index_proximal_start_x***	0.8024
***right_middle_proximal_start_x***	0.8024
***left_middle_proximal_start_x***	0.8023
***right_ring_proximal_start_x***	0.8021

**Table 6:** Results of the three trained models applied to unseen data.

Approach	Correct/Incorrect	Classification Accuracy
***RGB***	1250/1800	69.44%
***Leap Motion***	752/1800	41.78%
***Multi-modality***	**1377/1800**	**76.5%**

**Table 7:** Results for the models when trained via leave-one-subject-out validation. Each subject column shows the classification accuracy of that subject when the model is trained on the other four.

Model	Subject Left Out Accuracy (%)					Mean	Std.
	*1*	*2*	*3*	*4*	*5*		
***RGB***	81.12	68.24	93.82	89.82	94.15	85.43	9.79
***Leap Motion***	89.21	88.85	86.97	89.27	88.54	88.57	0.84
***Multi-modality***	85.52	96.7	87.51	93.82	97.1	92.12	4.76

**Table 8:** Results of pre-training and classification abilities of ASL models, with and without weight transfer from the BSL models.

Model	Non-Transfer from BSL		Transfer from BSL	
	*Epoch 0*	*Final Ability*	*Epoch 0*	*Final Ability*
***RGB***	2.98	80.68	13.28	81.82
***Leap Motion***	5.12	67.82	7.77	70.95
***Multi-modality***	5.12	65.4	21.31	82.55
